# Modulation of haematopoiesis by protozoal and helminth parasites

**DOI:** 10.1111/pim.12975

**Published:** 2023-02-28

**Authors:** Kyle T. Cunningham, Kingston H. G. Mills

**Affiliations:** ^1^ Wellcome Centre for Integrative Parasitology Institute of Infection and Immunity, University of Glasgow Glasgow UK; ^2^ Immune Regulation Research Group Trinity Biomedical Sciences Institute, Trinity College Dublin Dublin Ireland

**Keywords:** haematopoiesis, immune modulation, immunological terms, innate immunity, parasite

## Abstract

During inflammation, haematopoietic stem cells (HSCs) in the bone marrow (BM) and periphery rapidly expand and preferentially differentiate into myeloid cells that mediate innate immune responses. HSCs can be directed into quiescence or differentiation by sensing alterations to the haematopoietic niche, including cytokines, chemokines, and pathogen‐derived products. Most studies attempting to identify the mechanisms of haematopoiesis have focused on bacterial and viral infections. From intracellular protozoan infections to large multicellular worms, parasites are a global health burden and represent major immunological challenges that remain poorly defined in the context of haematopoiesis. Immune responses to parasites vary drastically, and parasites have developed sophisticated immunomodulatory mechanisms that allow development of chronic infections. Recent advances in imaging, genomic sequencing, and mouse models have shed new light on how parasites induce unique forms of emergency haematopoiesis. In addition, parasites can modify the haematopoiesis in the BM and periphery to improve their survival in the host. Parasites can also induce long‐lasting modifications to HSCs, altering future immune responses to infection, inflammation or transplantation, a term sometimes referred to as central trained immunity. In this review, we highlight the current understanding of parasite‐induced haematopoiesis and how parasites target this process to promote chronic infections.

## 1 INTRODUCTION TO HOMEOSTATIC AND INFECTION‐INDUCED HAEMATOPOIESIS

Under homeostatic conditions, there is a constant renewal of red and white blood cells. Hundreds of billions of new cells are produced each day in the bone marrow (BM) of humans.^1^ This process, known as haematopoiesis, is essential for steady‐state function and maintenance. During inflammation, local cells that recognize pathogen‐associated molecular patterns (PAMPs) and damage‐associate molecular patterns (DAMPs) become activated and either send signals or traffic to the BM to initiate emergency haematopoiesis. This allows for the rapid and efficient production of new immune cells which traffic to the site of inflammation. Haematopoiesis is essential for the robust induction of immune responses. Because of their short half‐life, innate immune cells require constant renewal. For example, monocytes, which patrol the periphery and differentiate into effector macrophages and dendritic cells (DCs), have a steady state half‐life of approximately 2 days.^2^ Homeostatic and emergency haematopoiesis are dependent on numerous factors, including cell–cell interactions in the haematopoietic niche, cytokines, metabolic changes, and BM‐invading pathogens.^3^, ^4^

Haematopoiesis primarily occurs in the BM, where haematopoietic stem cells (HSCs) differentiate from oligo‐ and multi‐potent progenitor cells into mature effector cells. Notably, this process has also been shown to occur in other organs, including the spleen, gastrointestinal tract, liver and the brain. Haematopoiesis in the BM occurs in a highly specialized compartment. The haematopoietic niche is tightly controlled by a collection of immune cells, stromal cells, mesenchymal stem cells, neuronal synapses, and a small number of blood vessels. This niche provides a buffer for the HSCs from the environment, inhibiting the induction of aberrant haematopoiesis during steady state, while promoting controlled emergency haematopoiesis in response to inflammation.^5^

Differentiation of HSCs follows a loose hierarchical structure, with long‐lived oligopotent HSCs becoming multipotent progenitors, followed by lineage‐committed progenitor cells, and finally mature immune cells. In mammals, the long‐term HSC (LT‐HSC) resides at the top of this haematopoiesis hierarchy (Table 1). LT‐HSC are primarily quiescent, long‐lived cells defined by their ability to self‐renew and differentiate into every cell of the haematopoietic cell system.^6^, ^7^, ^8^, ^9^ To respond to the haematopoietic needs of the body, LT‐HSCs express multiple receptors, including receptors for interleukin (IL)‐1, IL‐6, tumour necrosis factor (TNF), interferons (IFNs), and colony‐stimulating factors (CSFs).^9^, ^10^ Upon activation, LT‐HSCs differentiate into short‐term (ST)‐HSCs and multipotent progenitors (MPPs). During steady state and emergency haematopoiesis, MPPs are responsible for the majority of haematopoiesis.^9^, ^11^ However, both LT‐ and ST‐HSCs have been shown to promote haematopoiesis in homeostatic conditions, highlighting the complexity of this process.^12^, ^13^, ^14^

**TABLE 1:** HSC subpopulations and lineage potential.

Cell population	Surface markers	Lineage potential
LT‐HSC	Lin^−^ CD45^+^ cKit^+^ Sca^‐^1^+^ CD135^−^ CD48^−^ CD150^+^	Oligopotent
ST‐HSC	Lin^−^ CD45^+^cKit^+^ Sca^‐^1^+^ CD135^−^ CD48^+^ CD150^+^	Oligopotent
MPP	Lin^−^ CD45^+^ cKit^+^ Sca^‐^1^+^ CD135^−^ CD48^+^ CD150^−^	Oligopotent
MPP3	Lin^−^ CD45^+^ cKit^+^ Sca^‐^1^+^ CD135^−^ CD48^+^ CD150^−^ CD34^+^ CD135^−^	Oligopotent, myeloid bias
MPP4	Lin^−^ CD45^+^ cKit^+^ Sca^‐^1^+^ CD135^−^ CD48^+^ CD150^−^ CD34^+^ CD135^+^	Oligopotent, lymphoid bias

*Note*: Lineage negative (Lin^−^): CD11b^−^CD3^−^CD19^−^Ly6G^−^Siglec‐F^−^Ter119^−^.

Abbreviations: HSCs, haematopoietic stem cells; LT, long‐term; MPP, multipotent progenitor; ST, short‐term.

Although initially described as a homogenous collection of multipotent cells, single‐cell transcriptomic, and epigenomic sequencing has revealed that MPPs are a heterogenous population of four small subtypes (MPP1‐4).^15^, ^16^ Each of these MPP subtypes can differentiate into every haematopoietic cell, however, each MPP subset has a defined transcriptional bias towards a preferred lineage. For example, MPP4, also known as lymphoid MPPs, display a higher probability of differentiating into lymphoid lineage cells, whereas MPP2 and MPP3 are significantly more likely to develop into myeloid cells.^11^ This transcriptional bias is shown in the expression of various lineage‐specific gene transcripts, however, there is significant overlap in expression between subtypes. After activation with various inflammatory factors, MPPs further differentiate into the lineage‐committed progenitors, the common myeloid progenitor (CMP), and the common lymphoid progenitor (CLP). In turn, these lineage‐committed progenitors differentiate into increasingly cell‐specific precursors, before finally developing into mature immune cells.

Despite their sequestration in the BM niche, HSCs and progenitor cells express receptors for cytokines, growth factors, neurotransmitters, and chemokines. Interestingly, these oligopotent progenitors also express pattern recognition receptors (PRRs) and can respond to PAMPs, such as lipopolysaccharide (LPS) binding to toll‐like receptor (TLR) 4.^17^ TLR agonists have been shown to directly activate HSCs and induce their proliferation and differentiation in vitro and in vivo. Zhang et al. demonstrated that a single intraperitoneal injection of mice with flagellin, a TLR5 and NLR4 agonist, induced proliferation of HSCs.^18^ HSCs from flagellin‐injected mice showed improved engraftment when transferred to irradiated mice, with a preference for myelopoiesis.

During infection, these signals coalesce to induce adaptive responses in the BM niche that direct appropriate differentiation of mature immune cells. For example, infection with the bacterial pathogen *Listeria monocytogenes* induces signals in the HSC niche that induce the proliferation and expansion of monocytes.^19^ In addition, recent research into emergency haematopoiesis has found that IFNs play a key role in activating and modulating HSCs in the control of bacterial infections. For example, in mice infected with *Mycobacterium avium*, HSCs respond to IFN‐γ signalling to proliferate and increase myelopoiesis.^20^ In contrast, type I IFNs have a negative influence on myelopoiesis, whereas type II IFNs provide protection in mice infected with *Mycobacterium tuberculosis*. IFN can include long‐lasting modifications to HSCs, with altered phenotypes persisting for up to 1‐year.^21^

Parasites infect hundreds of millions of people and are a major health burden across the world. Parasites represent a diverse group, ranging from unicellular protozoan parasites, such as the *Plasmodium* species responsible for malaria, to multi‐cellular parasitic worms, also known as helminths, such as *Fasciola hepatica* or liver fluke. In general, infection with intracellular parasites induces type 1 immune responses, whereas helminths induce type 2 immune responses. However, parasites, especially helminths also actively modulate these immune responses. The induction and modulation of peripheral immune responses by parasites have been extensively studied and reviewed in the context of both protozoan and helminth parasites.^22^, ^23^, ^24^, ^25^, ^26^ Parasite infections are typically chronic and can alter the host in a myriad of ways. During acute infections with bacterial and viral agents, HSCs respond via transient activation and differentiation, followed by a return to quiescence. However, chronic infections with parasites, and the corresponding long‐lasting cytokine stimulation can lead to HSC exhaustion and dysfunction. Haematopoiesis in response to acute and chronic bacterial, viral, and fungal infections has been well characterized and documented.^27^, ^28^, ^29^ There is more limited knowledge on the influence of parasitic infections on haematopoiesis. In this review, we highlight the emerging evidence of a role for haematopoiesis in expanding anti‐parasite immune responses, and how parasites have evolved to promote dysfunctional haematopoiesis to promote their survival in the host.

## 2 PROTOZOAN PARASITE INFECTION AND HAEMATOPOIESIS

Protozoa are a large family of unicellular organisms, which includes *Plasmodium*, *Leishmania*, *Toxoplasma*, and *Trypanosoma* species. Protozoan infections are typically spread through the faecal‐oral route or by arthropod vectors, such as sand flies or mosquitos. There are different treatment approaches for different species, however, persistent, or recurrent infections are common. A robust immune response is essential for survival after infection with a protozoan parasite, which is often lethal in the immunocompromised individuals. Early responding immune cells, in particular neutrophils, monocytes, and macrophages, migrate to the site of infection and mediate characteristic type 1 immune responses. This includes classical activation of macrophages, and production of pro‐inflammatory mediators, such as IFN‐γ, IL‐1β, IL‐12, and nitric oxide.^23^, ^30^ To maintain effective immune responses, innate immune cells, both tissue‐resident and infiltrating, require constant renewal from HSCs. This can occur centrally in the BM, or in the periphery at the site of inflammation, otherwise known as in situ haematopoiesis. For example, Osorio et al. show that *Leishmania* infection enhances local proliferation of infiltrating monocytes in the spleens of infected hamsters.^31^ However, much like viral and bacterial pathogens, protozoan species have adapted to manipulate this emergency haematopoiesis to benefit their own survival. Locally proliferating monocytes, which contribute to the characteristic splenomegaly of leishmaniasis, were found to be preferred targets for infiltrating parasites. Targeting the in situ haematopoiesis with STAT3 inhibitors decreased monocyte proliferation and parasite burden.^31^

### 2.1 Leishmania

In contrast to large parasitic worms, single‐celled parasites are uniquely capable of directly altering haematopoiesis in the BM. Indeed, many protozoan parasites, including *Leishmania* species can invade the BM or act on peripheral HSCs. *Leishmania* parasites are spread via sandflies and are found throughout the world, the two dominant species being *Leishmania donovani* and *L. chagasi*.^32^ Once inside the mammalian hosts, the *Leishmania* parasites primarily reside in monocyte‐derived macrophages and DCs in the liver and spleen. However, it has also been widely reported that *Leishmania* can be found in the BM of infected individuals.^32^ Indeed, a study of Brazilian patients suffering from visceral leishmaniasis, found that an increased number of amastigotes in the BM correlated with increased parasite load in the periphery.^33^ Furthermore, patients who died from the infection had high parasite burdens in the BM. *Leishmania* activate the HSC compartment in the BM to promote the expansion of select immune cells. For example, infection of mice with *L. donovani* induces IFN‐γ‐secreting CD4^+^ T cells in the BM, which direct development and activation of monocytes.^34^

BM‐resident macrophages can act as a reservoir of *Leishmania* parasites. During infection with *L. donovani* BM‐resident macrophages are susceptible to infection and promote the expansion of HSC and myelopoiesis through secretion of granulocyte‐macrophage CSF (GM‐CSF) and TNF.^35^, ^36^ *L. donovani* has also been shown to specifically direct HSCs to differentiate into monocytes that are more permissible to infection.^37^ Monocytes found in the BM of infected mice had a regulatory phenotype and a reduced capacity to differentiate into macrophages capable of initiating pro‐inflammatory anti‐leishmania responses. Moreover, this was mediated by cytokines, such as IL‐1β and IFN‐γ, and various growth factors in the BM. In support of these findings, it was demonstrated that BM extracellular fluid from infected mice directly expanded HSCs and generated suppressive monocytes seen during experimental infections.^37^ In a separate study, HSCs from *L. donovani*‐infected mice were found to have defective self‐renewal and engraftment abilities. This coincided with increased proliferation of LT‐HSCs, mediated by TNF‐induced expansion and maintenance of IFN‐γ‐secreting BM‐resident CD4^+^ T cells.^38^ Mice lacking T and B cells showed no changes to their HSC populations following infection with *L. donovani*, despite displaying an increase in parasite burden. Modulation of HSCs appears to occur across multiple *Leishmania* species, as experimental cutaneous infection of mice with *L. major* induces the expansion of LT‐HSC in BM, and myeloid‐biased MPP2/3 in the BM and spleen.^39^

Despite the evidence that mature immune cells and stromal cells are the predominant hosts for *Leishmania* in the BM, it has recently been reported that HSC harboured the parasites in Syrian hamsters infected with *L. donovani*.^36^, ^40^, ^41^ Using bioluminescent live imaging, *Leishmania* were found in the BM during infection, as well as post‐treatment with anti‐leishmanial drugs. After treatment, *Leishmania* in the BM were found to repopulate the spleen and liver, therefore identifying the BM as an essential niche for parasite persistence. Crucially, while *Leishmania* have previously been shown to reside in BM‐resident mature immune cells, this study demonstrated that long‐lived LT‐HSC also provides a key sanctuary for the parasite during leishmania infection.

### 2.2 Plasmodium

In humans, malaria is primarily caused by *Plasmodium falciparum* and *P. vivax*.^42^ Like *Leishmania*, *Plasmodium* species have been shown to reside in the BM.^43^, ^44^, ^45^ Interestingly, a recent study of patients found that total parasite biomass in the BM, rather than in the blood, correlated strongly with poor prognosis.^46^ Histological and genetic screening of samples collected from children who died from *P. falciparum* in Malawi found that the BM contained a large reservoir of parasites.^47^ Further histological analyses revealed that the parasites were located primarily extravascularly, with many residing in BM macrophages, and some found directly adjacent to erythroid progenitors.

A number of studies have attempted to decipher the mechanisms of *Plasmodium*‐induced alterations to haematopoiesis. Belyaev et al. found that mice infected with *P. chabaudi* had increased numbers of IL‐7 receptor‐expressing lymphoid‐biased MPPs in the BM by day 11 post‐infection, and this was dependent on IFN‐γ signalling in haematopoietic progenitors.^48^ Upon isolation and culture ex vivo, these malaria‐induced lymphoid‐biased MPPs lacked erythroid potential and preferentially differentiated into lymphoid and myeloid cells. Similarly, *P. berghei* infection of mice expands the oligopotent HSC populations in the BM via a mechanism that involved IFN‐γ and IL‐27.^49^ These cytokines aided in expanding HSCs in the BM, followed by HSC mobilization to the spleen and induction of rapid myelopoiesis to resolve the infection.

As *Plasmodium* is found beyond the BM, extramedullary, or non‐BM, haematopoiesis is essential to fight the infection. Like *P. berghei*, mice infected with *P. yoelii* display a rapid and sustained increase of progenitor populations in the spleen.^50^, ^51^ Interestingly, these progenitor cells were essential for establishing splenic immune responses to the parasite through secretion of IL‐17 and activation of local stromal cell populations. The activated stromal cells promoted B‐cell differentiation in vitro when co‐cultured with the expanded splenic progenitors. Further studies have demonstrated the importance of extramedullary haematopoiesis in plasmodium infections. During infection with *P. chabaudi*, mice have fewer myeloid‐committed progenitors, CMP and GMP, in the BM, while the number of these progenitors increase in the spleen.^52^ This was dependent on IFN‐γ‐induced increases in serum CXCL10 and CCL2, which caused mobilization of the CCR2^+^ CMP and GMP to the spleen.

### 2.3 Toxoplasma

*Toxoplasma gondii* is a protozoan parasite that infects millions of people around the world. Toxoplasmosis induces a strong type 1 response, which is essential for pathogen elimination and host survival. Indeed, mice lacking IFN‐γ or IL‐12 are highly susceptible to *Toxoplasma* infections.^23^, ^30^, ^53^ However, unlike *Leishmania* and *Plasmodium* infections, many people are undiagnosed and live asymptomatically with the *Toxoplasma* parasite. In a study of patients with chronic *T. gondii*, peripheral blood monocytes had significantly altered surface expression of various markers, including immunoglobulin receptors and MHC molecules.^54^ Furthermore, IL‐12 mRNA expression was enhanced in monocytes from patients following ex vivo stimulation with *Toxoplasma*. Since monocytes have a short lifespan, these alterations could potentially be due to infection‐associated modifications to the HSC population. *Toxoplasma* can invade haematopoietic and non‐haematopoietic cells in the BM. However, due to its global range, and often asymptomatic disease, toxoplasma can go undiagnosed and present potentially fatal risks for patients, in particular those receiving BM transplants.^55^, ^56^, ^57^, ^58^, ^59^ Lopes et al., have shown that infection of BM donor mice with two different strains of *T. gondii* resulted in decreased survival of recipient mice.^60^ *T. gondii* drastically alters erythropoiesis and skews HSCs towards granulopoiesis which expands effector immune cells that fight infection.^23^, ^30^ This was recently found to be mediated by the induction of systemic IL‐6 signalling, leading to a reduction in erythrocyte progenitors and a subsequent increase in the GMP population in the BM of infected mice.^61^ Importantly, this shift away from erythropoiesis was found to be exclusive to the BM; splenic erythropoiesis was unaffected, highlighting the role of extramedullary haematopoiesis in response to infection.

### 2.4 Trypanosoma

*Trypanosoma* species are found throughout the world, although the two most common species *Trypanosoma cruzi* and *T. brucei*, the causative agents of Chagas disease and African sleeping sickness, respectively, are found in South America and Africa. Similar to other single‐cell parasites, trypanosomes can infiltrate the BM. Indeed, stromal cells infected with the parasite are found 2 weeks post‐challenge in mice infected with *T. cruzi*.^62^ Parasite infiltration of the BM coincided with an increase in HSC populations, including MPP3/4 and CMPs, with an accompanying decrease in lymphoid‐biased MPPs and CLP. Interestingly, the authors also found a concomitant increase in HSCs in the spleens of infected mice, highlighting again the importance of extramedullary haematopoiesis in counteracting impairments in the BM.

Infection of mice with experimental and field isolates of Trypanosome strains, *T. brucei* and *T. congolense*, can rapidly deplete B cells in the BM and spleen.^63^, ^64^, ^65^ Infection of mice with *T. cruzi* has been shown to infect BM stromal cells, leading to a reduction in IL‐7, an essential cytokine for the differentiation of B cells from progenitor cells.^66^ Indeed, reduction in mature B cells has been shown to coincide with severe depletion of up to 95% of CLP, pre‐ and pro‐B cell precursors, and immature B cells in the BM of mice infected with T*. brucei*.^63^ This depletion coincided with an increase in compensatory B‐cell lymphopoiesis in spleens, as indicated by increased numbers of B‐cell precursors and HSCs. In addition to impaired B‐cell lymphopoiesis, whole BM from *T. cruzi*‐infected mice have a significantly reduced ability to differentiate across many lineages, including myeloid and erythroid, an effect that was transferrable to naïve mice via BM transplantation.^67^ However, this is effectively prevented by treatment with anti‐trypanosome therapeutics. Furthermore, a recent study demonstrated that treatment with commercially available anti‐trypanosome drugs increased the total number of B‐cell precursors in the BM of *T. congolense*‐infected mice.^63^

**FIGURE 1:**
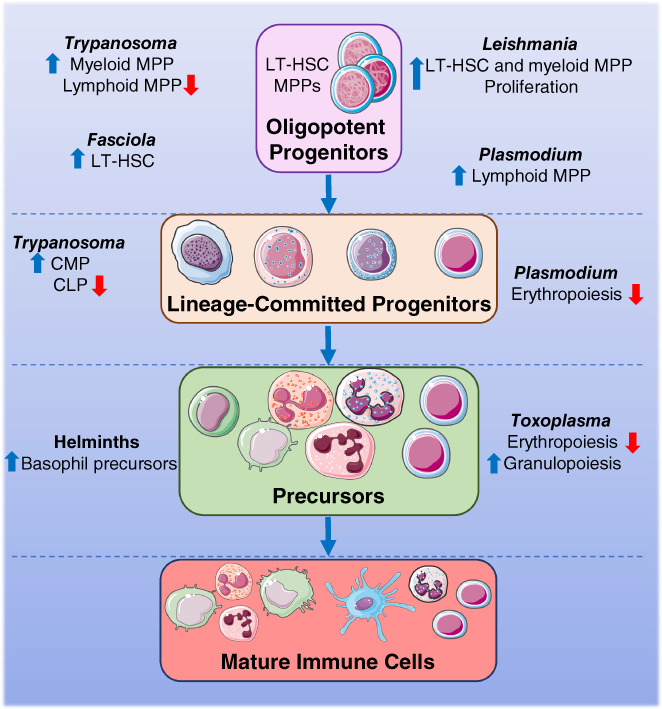
Parasites target HSCs throughout haematopoiesis. Simplified hierarchical view of haematopoiesis, beginning with oligopotent progenitors (LT‐HSC, MPP), followed by lineage‐committed progenitors (CMP, CLP, MEP), then direct precursors (e.g., basophil precursor, MDP, cMoP), and finally mature immune cells. Many parasites modulate this pathway directly and indirectly to improve their survival in the host. This includes, increasing proliferation, enhanced differentiation, or inhibition of development of specific lineages. CLP, common lymphoid progenitor; cMoP, common monocyte precursor; CMP, common myeloid progenitor; HSCs, haematopoietic stem cells; LT‐HSC, long‐term HSC; MDP, macrophage/dendritic cell precursor; MEP, megakaryocyte/erythrocyte progenitor; MPP, multipotent progenitor.

Long‐lasting disruption to B‐cell lymphopoiesis induced by *Trypanosoma* species has serious implications for the generation of immune responses to subsequent infections and vaccinations. A recent study demonstrated that infection with *T. brucei* after immunization with an acellular pertussis vaccine abolished the protective effects of the vaccine against *Bordetella pertussis* infection.^68^ The *T. brucei* infection post‐vaccination was cleared with an anti‐parasite drug and mice were intranasally challenged with *B. pertussis* 8 weeks. Mice that had been infected with *T. brucei* prior to *B. pertussis* challenge were unable to mount competent B‐cell responses to the bacteria and subsequently were unable to clear the infection from the lung. Conversely, a study that assess the efficacy of *T. brucei*‐induced killing of B cells in a mouse model of multiple myeloma showed that infected mice survived significantly longer than un‐infected mice, in part due to the potent suppression of B‐cell lymphopoiesis.^69^ Collectively, these reports demonstrate that protozoan parasites can invade the BM and modulate haematopoiesis, which impacts the host immune responses to other diseases (Figure 1).

## 3 PARASITIC WORMS AND HAEMATOPOIESIS

Parasitic worms or helminths, have evolved mechanisms of suppressing host immune responses to prevent their ejection and thereby maintain long‐term infections. The host immune response has also co‐evolved with parasite to limit infection‐associated pathology. Infection with helminths induces a robust type 2 response, characterized by prominent Th2 cells, eosinophils, alternatively activated macrophages, mast cells, basophils, and innate lymphoid type 2 cells (ILC2s).^25^ Helminths have developed sophisticated methods of immune modulation to promote chronic infection, including induction of regulatory and anti‐inflammatory immune responses.^25^, ^70^, ^71^ The size and life cycles of helminths, which often include migration through the body and damage to the host, can result in significant immune activation. This mechanical stress, as well as to excreted/secreted factors (ES) produced by the parasites induce release of type 2 alarmins, including IL‐25, IL‐33, and TSLP. These cytokines activate local immune cell populations, induce differentiation of HSCs in the BM, and promote in situ haematopoiesis, for example during infection with the blood fluke Schistosoma mansoni.^72^, ^73^

Although not yet fully understood, modifications to BM HSC have been demonstrated during helminth infections. Transplantation of BM from mice susceptible to helminth infection into normally resistant recipient mouse strains can transfer susceptibility to infection.^74^ The transfer of susceptibility was specific to the migratory, rather than the tissue, stage of infection with *Nippostrongylus brasiliensis*, suggesting a role for emergency haematopoiesis and production of new circulating immune cells. Babayan et al. found that infection of mice with the filarial nematode *Litomosomoides sigmodontis* enhanced multipotent progenitor populations in the BM of aged compared with young mice and this correlated with an increase in worm burden.^75^ Furthermore, stimulation of HSCs with the filarial nematode product ES‐62 promoted the differentiation of tolerogenic DCs which were hyporesponsive to LPS stimulation and promoted induction of Th2 cells.^76^

Infection with the helminths *Heligmosomoides polygyrus* and *N. brasiliensis*, used extensively as mouse models of helminth infection, is associated with expansion of basophils in the BM and spleen. *H. polygyrus* infection induced the expansion of basophil precursors in the BM through the combined effect of IL‐3, IL‐4, and immunoglobulins.^77^ IL‐33, released upon mechanical stress, has been shown to indirectly promote the expansion and differentiation of basophils through induction of IL‐3 and GM‐CSF.^78^ During infection with *Trichuris muris*, a mouse model of the human roundworm *Trichuris trichiura*, TSLP acts directly on precursors in the BM to induce basophil expansion.^79^ TSLP can directly induce extramedullary haematopoiesis, expanding splenic GMP‐like cells capable of differentiating into macrophages, dendritic cells, and granulocytes.^80^ Transfer of the TSLP‐induced GMP‐like cells into TSLP receptor deficient mice infected with *T. muris* resulted in improved goblet cell hyperplasia, enhanced type 2 cytokine production and a significantly lower parasite burden. Infections with *T. muris* or *N. brasiliensis* induce IL‐25‐dependent increases in a type 2 MPP in the gastrointestinal tract‐associated lymphoid tissues.^81^ Interestingly, the increased type 2 MPPs generated by the helminth infections were not found in the spleen or BM. Furthermore, lineage mapping studies showed that these cells were able to differentiate into mast cells, basophils, and macrophages in response to type 2 cytokines. Subsequent studies showed that IL‐33 and IL‐25 were responsible for enhancing ILC2s and the type 2 MPPs in the mesenteric lymph nodes, lungs, and blood.^82^ Moreover, when adoptively transferred into mice lacking the IL‐25 receptor, type 2 MPPs differentiated and induced IgE and goblet cell hyperplasia to promote clearance of *T. muris* infection. Although typically expelled quickly via type 2 inflammation in acute infection, chronic infection with *T. muris*, initiated by low‐dose infection, induces expansion and proliferation of oligopotent HSCs and neutrophils in the BM.^83^ Interestingly, no changes occur to the HSC compartment during acute infections, which are cleared rapidly. Instead, chronic *T. muris* induces an ineffective type 1 response and expansion of IFN‐γ‐secreting CD4^+^ and CD8^+^ T cells in the BM, resulting in proliferation of LT‐HSC and MPP, culminating in neutrophil expansion and worm persistence.

Infection of mice with the nematode parasite *Trichinella spiralis*, the parasite responsible for trichinosis, is characterized by rapid expansion of mast cells and basophils from the BM, which migrate to tertiary lymphoid organs, such as the spleen and mesenteric lymph nodes, and to the site of infection.^84^, ^85^ During early infection with *T. spiralis*, the lineage‐committed progenitors CLP and GMP are significantly expanded, while oligopotent LT‐HSCs and MPPs are unchanged.^86^ The study utilized intravital microscopy to show that *T. spiralis* infection induced a dramatic mobilization, as well as expansion, of HSC. HSCs in infected mice remain largely in their niche, with approximately 12% undergoing some form of movement, however, *T. spiralis* infection promoted expansion and migration of nearly 60% of HSC from the BM. Furthermore, transplantation of mixed chimera BM from uninfected and infected mice showed that BM from *T. spiralis*‐infected mice had short‐ and long‐term advantages in repopulating irradiated recipient mice. The study of *T. spiralis* infection has also led to the discovery of a common erythrocyte‐mast cell progenitor population in the HSC compartment.^87^, ^88^ Using single‐cell RNA sequencing, Inclan‐Rico and colleagues found that erythrocyte and mast cells progenitors exist at steady state in the BM, spleen, and lymph nodes. However, during *T. spiralis* infection, these HSCs are activated and mobilized to leave the BM and expand in the spleen and mesenteric lymph nodes where they differentiate and replenish mast cells at the site of infection.

In addition to haematopoiesis in the BM, stems cells in the intestine can, under the influence of IL‐4 and IL‐13, expand and differentiate into the secretory tuft and goblet cells required for worm clearance from the gut.^89^, ^90^, ^91^ Mice lacking transcription factors involved in tuft cell differentiation from resident intestinal stem cells were unable to clear infection with *N. brasiliensis*.^90^ Indeed, during *N. brasiliensis* infection, newly differentiated tuft cells secrete IL‐25, which in turn activates local ILC2s, which initiates a feed‐forward loop to promote proliferation and expansion of tuft cells and protective type 2 immune responses.^89^, ^90^, ^92^ This pathway has been targeted by helminths to enable them to evade expulsion. *H. polygyrus* infection induces activation and proliferation of intestinal stem cells, however, they have significantly impaired ability to differentiate into tuft cells.^93^, ^94^ Ex vivo stimulation of intestinal organoids (three‐dimensional structures that mimic the murine intestine) with ES from *H. polygyrus* (HES) induces proliferation and expansion of stem cells, creating spheroid‐like structures.^93^ Interestingly, stimulation with HES, or co‐culture of with larvae, inhibited IL‐4 and IL‐13 induced differentiation of stem cells into tuft cells. Furthermore, co‐infection with *H. polygyrus* and *N. brasiliensis* significantly reduced *N. brasiliensis*‐induced expansion of tuft cells in the gut epithelium. Collectively these studies demonstrate that helminths can modulate haematopoiesis in the BM but also differentiation of stem cells in the intestine (Figure 1).

## 4 CENTRAL TRAINED INNATE IMMUNITY DURING PARASITE INFECTION

**FIGURE 2:**
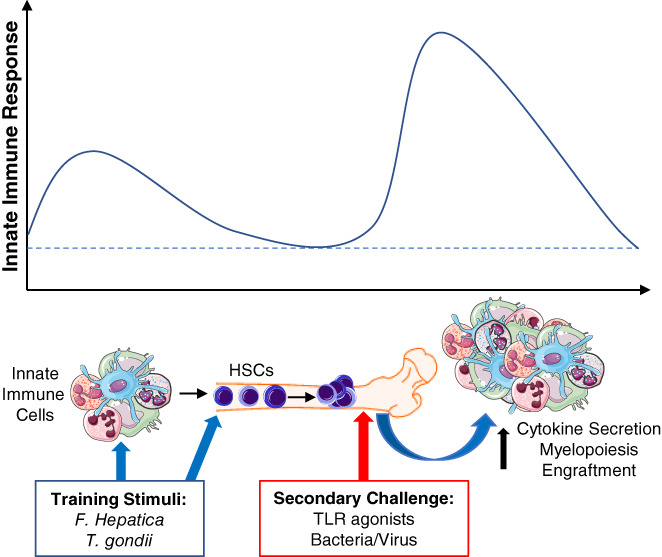
Central trained innate immunity and parasitic infections. Infection with parasite or administration of parasite‐derived, such as *Fasciola hepatica* ES or *Toxoplasma gondii*, act on peripheral innate immune cells and haematopoietic stem cells (HSCs) to imprint epigenetic modifications that produce trained innate immune cells that respond to secondary stimuli with enhanced responses. Protozoan‐ and helminth‐induced changes to HSCs result in bias towards myelopoiesis and improved engraftment during transplantation. Epigenetic and metabolic reprogramming induces long‐term enhanced effector responses in future generations of innate immune cells, that can modulate development of autoimmunity.

Although initially thought to be exclusive to T and B cells, cells of the innate immune system can undergo training and display features of immunological memory. When primed with certain stimuli, innate immune cells undergo metabolic and epigenetic remodelling before returning to an inactive state. Upon reactivation with PAMPs or cytokines, these primed innate immune cells respond with enhanced effector capabilities.^95^, ^96^, ^97^ This process, termed trained innate immunity, has been demonstrated in NK cells, monocytes, macrophages, and dendritic cells.^96^ Trained innate immunity has primarily been studied with respect to patrolling and tissue‐resident mature innate immune cells. However, innate immune cells are largely short‐lived, while many studies have reported effects lasting many months, indicating mechanisms of long‐lasting modification to pools of innate immune cells or their progenitors. In recent years, certain pathogens, vaccines, and PAMPs have been reported to induce long‐term alterations to HSCs. This has been termed central trained immunity, where activation by certain stimuli, such as β‐glucan or *Mycobacterium tuberculosis*, induces metabolic and epigenetic rewiring of HSCs.^98^, ^99^ This rewiring, or training, is a type of non‐specific memory to inflammatory stimuli, allowing HSCs to respond faster and more acutely to secondary challenge.^100^ HSCs, including LT‐HSCs and MPPs, have been identified as targets of central trained immunity. For example, vaccination with BCG expands and modifies MPP, imprinting a bias towards myelopoiesis.^99^ Furthermore, macrophages expanded from these MPP have enhanced bacterial killing capacities and increase mRNA expression of pro‐inflammatory cytokines. Research on central trained immunity has largely focused on the modulatory effects of bacterial and fungal products, including LPS, β‐glucan or flagellin, or on pro‐inflammatory cytokines, such as IL‐1β and IFN‐γ.^18^, ^21^, ^99^, ^101^, ^102^, ^103^, ^104^ However, recent studies have demonstrated that central trained immunity can also be mediated by parasites (Figure 2).

Central trained immunity has been described following infection with the protozoal parasite *T. gondii*. Mice infected with *T. gondii* exhibit rapid expansion of monocytes along with increased pro‐inflammatory cytokine production.^105^ Askenase et al., found that during infection with *T. gondii*, IL‐12‐activates BM‐resident NK cells to produce IFN‐γ, which primes the common monocyte precursor (cMoP) and immature monocytes.^105^ These primed monocytes developed a regulatory phenotype following stimulation with various PAMPs, such as LPS and flagellin. Importantly, analysis of *T. gondii*‐primed monocyte gene expression via Nanostring revealed increased expression of both anti‐ and pro‐inflammatory genes after LPS stimulation, indicating a non‐specific augmentation of innate immune responses rather than selective enhancement of anti‐inflammatory response.

We have demonstrated that treatment of mice with ES from the helminth *F. hepatica* (FHES) imprints a long‐lasting memory on HSCs.^106^ Mice treated with FHES had enhanced proliferation and expansion of LT‐HSCs and myeloid‐committed precursors, resulting in expansion of anti‐inflammatory monocytes. This helminth‐induced anti‐inflammatory trained innate immunity rendered the mice less susceptible to induction of experimental autoimmune encephalomyelitis, a mouse model of multiple sclerosis. The protection against development of Th1‐ and Th17‐driven autoimmune disease persisted for up to 18 months after treatment with the helminth products and could be transferred to naïve mice via transplantation of LT‐HSCs isolated from FHES‐treated mice.

## 5 CONCLUSIONS

Immune responses to parasitic infections involve a plethora of immune and non‐immune cells, which activate and induce local and systemic modifications to aid in parasite expulsion. Most of the work to date has been done on mature immune cells. However, there is emerging evidence of roles for HSCs in combatting infections. HSCs play a fundamental role in shaping, as well as generating, the cells that mediate innate and adaptive immune responses that are mobilized to act at the site of infection. The HSC provide memory or trained cells of the innate, as well as adaptive, immune system also help to fight future infections and inflammation. Understanding how HSCs are activated and differentiated may aid in the discovery of novel therapeutics for various parasitic infections, but may also help to identify new targets for long‐lasting disease‐modifying treatments for autoimmune and other inflammatory diseases.

## AUTHOR CONTRIBUTIONS

Kyle T. Cunningham and Kingston H. G. Mills wrote the review.

## CONFLICT OF INTEREST STATEMENT

The authors declare no conflict of interest.

### PEER REVIEW

The peer review history for this article is available at https://publons.com/publon/10.1111/pim.12975.

## Data Availability

Data sharing is not applicable to this article as no new data were created or analyzed in this study.

## References

[1] Fliedner TM , Graessle D , Paulsen C , Reimers K . Structure and function of bone marrow hemopoiesis: mechanisms of response to ionizing radiation exposure. Cancer Biother Radiopharm. 2002;17(4):405‐426. 12396705 10.1089/108497802760363204

[2] Yona S , Kim KW , Wolf Y , et al. Fate mapping reveals origins and dynamics of monocytes and tissue macrophages under homeostasis. Immunity. 2013;38(1):79‐91. doi:10.1016/j.immuni.2012.12.001 23273845 PMC3908543

[3] Boettcher S , Gerosa RC , Radpour R , et al. Endothelial cells translate pathogen signals into G‐CSF‐driven emergency granulopoiesis. Blood. 2014;124(9):1393‐1403. 24990886 10.1182/blood-2014-04-570762 PMC4148762

[4] Fröbel J , Landspersky T , Percin G , et al. The hematopoietic bone marrow niche ecosystem. Front Cell Dev Biol. 2021;9:705410. 34368155 10.3389/fcell.2021.705410 PMC8339972

[5] Pinho S , Frenette PS . Haematopoietic stem cell activity and interactions with the niche. Nat Rev Mol Cell Biol. 2019;20:303‐320. 30745579 10.1038/s41580-019-0103-9 PMC6483843

[6] Cheng H , Zheng Z , Cheng T . New paradigms on hematopoietic stem cell differentiation. Protein and Cell. 2020;11:34‐44. doi:10.1007/s13238-019-0633-0 31201709 PMC6949320

[7] Zhang Y , Gao S , Xia J , Liu F . Hematopoietic hierarchy—an updated roadmap. Trends Cell Biol. 2018;28:976‐986. 29935893 10.1016/j.tcb.2018.06.001

[8] Cheshier SH , Morrison SJ , Liao X , Weissman IL . In vivo proliferation and cell cycle kinetics of long‐term self‐renewing hematopoietic stem cells. Proc Natl Acad Sci U S A. 1999;96:3120‐3125. 10077647 10.1073/pnas.96.6.3120 PMC15905

[9] Schoedel KB , Morcos MNF , Zerjatke T , et al. The bulk of the hematopoietic stem cell population is dispensable for murine steady‐state and stress hematopoiesis. Blood. 2016;128(19):2285‐2296. 27357698 10.1182/blood-2016-03-706010

[10] Borghesi L . Signals in hematopoietic stem cells conversion of danger signals into cytokine self‐renewal, lineage fate choice, and the hematopoiesis in steady‐state versus stress. J Immunol. 2018;193:2053‐2058. 10.4049/jimmunol.1400936 PMC4135530 25128551

[11] Pietras EM , Reynaud D , Kang Y‐A , Stuart JM , Gö B . Functionally distinct subsets of lineage‐biased multipotent progenitors control blood production in normal and regenerative conditions. Cell Stem Cell. 2015;17:35‐46. doi:10.1016/j.stem.2015.05.003 26095048 PMC4542150

[12] Sun J , Ramos A , Chapman B , et al. Clonal dynamics of native haematopoiesis. Nature. 2014;514(7522):322‐327. 25296256 10.1038/nature13824 PMC4408613

[13] Busch K , Klapproth K , Barile M , et al. Fundamental properties of unperturbed haematopoiesis from stem cells in vivo. Nature. 2015;518(7540):542‐546. 25686605 10.1038/nature14242

[14] Sawai CM , Babovic S , Upadhaya S , et al. Hematopoietic stem cells are the major source of multilineage hematopoiesis in adult animals. Immunity. 2016;45(3):597‐609. 27590115 10.1016/j.immuni.2016.08.007 PMC5054720

[15] Muller‐Sieburg CE , Cho RH , Karlsson L , Huang JF , Sieburg HB . Myeloid‐biased hematopoietic stem cells have extensive self‐renewal capacity but generate diminished lymphoid progeny with impaired IL‐7 responsiveness. Blood. 2004;103(11):4111‐4118. 14976059 10.1182/blood-2003-10-3448

[16] MacLean AL , Smith MA , Liepe J , et al. Single cell phenotyping reveals heterogeneity among hematopoietic stem cells following infection. Stem Cells. 2017;35(11):2292‐2304. 28833970 10.1002/stem.2692

[17] Nagai Y , Garrett KP , Ohta S , et al. Toll‐like receptors on hematopoietic progenitor cells stimulate innate immune system replenishment. 2018;24(6):801‐812. 10.1016/j.immuni.2006.04.008 PMC1626529 16782035

[18] Zhang B , Oyewole‐Said D , Zou J , Willliams IR , Gewirtz AT . TLR5 signaling in murine bone marrow induces hematopoietic progenitor cell proliferation and AIDS survival from radiation. Blood Adv. 2017;1(21):1796‐1806. 29296826 10.1182/bloodadvances.2017006981 PMC5728097

[19] Serbina N V , Hohl TM , Cherny M ., Pamer EG Selective expansion of the monocytic lineagedirected by bacterial infection. *J Immunol*. 2009;183(3):1900‐1910. 10.4049/jimmunol.0900612 PMC2753883 19596996

[20] Baldridge MT , King KY , Boles NC , Weksberg DC , Goodell MA . Quiescent haematopoietic stem cells are activated by IFN‐γ in response to chronic infection. Nature. 2010;465(7299):793‐797. 20535209 10.1038/nature09135 PMC2935898

[21] Khan N , Downey J , Sanz J , et al. *M. Tuberculosis* reprograms hematopoietic stem cells to limit myelopoiesis and impair trained immunity. Cell. 2020;183(3):752‐770.e22. 33125891 10.1016/j.cell.2020.09.062 PMC7599081

[22] Lopes MF , Zamboni DS , Lujan HD , Rodrigues MM . Immunity to protozoan parasites. J Parasitol Res. 2012;2012:250793. 22619699 10.1155/2012/250793 PMC3352617

[23] Gurung P , Kanneganti TD . Immune responses against protozoan parasites: a focus on the emerging role of nod‐like receptors. Cell Mol Life Sci. 2016;73(16):3035‐3051. 27032699 10.1007/s00018-016-2212-3 PMC4956549

[24] Silva‐Barrios S , Stäger S . Protozoan parasites and type I IFNs. Front Immunol. 2017;8(JAN):14. 28154565 10.3389/fimmu.2017.00014 PMC5243830

[25] Finlay CM , Walsh KP , Mills KHG . Induction of regulatory cells by helminth parasites: exploitation for the treatment of inflammatory diseases. Immunol Rev. 2014;259(1):206‐230. doi:10.1111/imr.12164 24712468

[26] Loukas A , Maizels RM , Hotez PJ . The yin and yang of human soil‐transmitted helminth infections. Int J Parasitol. 2021;51(13–14):1243‐1253. 34774540 10.1016/j.ijpara.2021.11.001 PMC9145206

[27] Caiado F , Pietras EM , Manz MG . Inflammation as a regulator of hematopoietic stem cell function in disease, aging, and clonal selection. J Exp Med. 2021;218(7):e20201541. 34129016 10.1084/jem.20201541 PMC8210622

[28] Zhang CC . Hematopoietic stem cells: interplay with immunity. Am J Blood Res. 2012;2(4):219‐227. 23226622 PMC3512180

[29] Sezaki M , Hayashi Y , Wang Y , Johansson A , Umemoto T , Takizawa H . Immuno‐modulation of hematopoietic stem and progenitor cells in inflammation. Front Immunol. 2020;11:585367. 33329562 10.3389/fimmu.2020.585367 PMC7732516

[30] Sasai M , Pradipta A , Yamamoto M . Host immune responses to toxoplasma gondii. Int Immunol. 2018;30(3):113‐119. 29408976 10.1093/intimm/dxy004

[31] Osorio EY , Medina‐Colorado AA , Travi BL , Melby PC . In‐situ proliferation contributes to the accumulation of myeloid cells in the spleen during progressive experimental visceral leishmaniasis. PLoS One. 2020;15(11):e0242337. 33180876 10.1371/journal.pone.0242337 PMC7660562

[32] Poulaki A , Piperaki ET , Voulgarelis M . Effects of visceralising *Leishmania* on the spleen, liver, and bone marrow: a pathophysiological perspective. Microorg. 2021;9(4):759. 10.3390/microorganisms9040759 PMC8066032 33916346

[33] Silva JM , Zacarias DA , De Figueirêdo LC , et al. Bone marrow parasite burden among patients with New World kala‐azar is associated with disease severity. Am J Trop Med Hyg. 2014;90(4):621‐626. 24615127 10.4269/ajtmh.13-0376 PMC3973504

[34] Romano A , Brown N , Ashwin H , et al. Interferon‐γ‐producing CD4+ T cells drive monocyte activation in the bone marrow during experimental *Leishmania donovani* infection. Front Immunol. 2021;12:700501. 34557190 10.3389/fimmu.2021.700501 PMC8453021

[35] Cotterell SEJ , Engwerda CR , Kaye PM . Enhanced hematopoietic activity accompanies parasite expansion in the spleen and bone marrow of mice infected with *Leishmania donovani* . Infect Immun. 2000;68(4):1840‐1848. 10722572 10.1128/iai.68.4.1840-1848.2000 PMC97356

[36] Cotterell SEJ , Engwerda CR , Kaye PM . *Leishmania donovani* infection of bone marrow stromal macrophages selectively enhances myelopoiesis, by a mechanism involving GM‐CSF and TNF‐alpha. Blood. 2000;95(5):1642‐1651. 10688819

[37] Abidin BM , Hammami A , Stä Ger S , Heinonen KM . Infection‐adapted emergency hematopoiesis promotes visceral leishmaniasis. 2017;13:e1006422. doi:10.1371/journal.ppat.1006422 PMC5560750 28787450

[38] Pinto AI , Brown N , Preham O , Doehl JSP , Ashwin H , Kaye PM . TNF signalling drives expansion of bone marrow CD4+ T cells responsible for HSC exhaustion in experimental visceral leishmaniasis. PLoS Pathog. 2017;13(7):e1006465. 28671989 10.1371/journal.ppat.1006465 PMC5510901

[39] Ferreira FLB , Séguin O , Descoteaux A , Heinonen KM . Persistent cutaneous *Leishmania* major infection promotes infection‐adapted myelopoiesis. Microorganisms. 2022;10(3):535. 35336108 10.3390/microorganisms10030535 PMC8954948

[40] Kaye P , Scott P . Leishmaniasis: complexity at the host–pathogen interface. Nat Rev Microbiol. 2011;9(8):604‐615. 21747391 10.1038/nrmicro2608

[41] Dirkx L , Hendrickx S , Merlot M , et al. Long‐term hematopoietic stem cells as a parasite niche during treatment failure in visceral leishmaniasis. Commun Biol. 2022;5(1):626. 35752645 10.1038/s42003-022-03591-7 PMC9233693

[42] Venugopal K , Hentzschel F , Valkiūnas G , Marti M . *Plasmodium* asexual growth and sexual development in the haematopoietic niche of the host. Nat Rev Microbiol. 2020;18(3):177‐189. 31919479 10.1038/s41579-019-0306-2 PMC7223625

[43] Obaldia N , Meibalan E , Sa JM , et al. Bone marrow is a major parasite reservoir in *Plasmodium vivax* infection. MBio. 2018;9(3):e00625‐18. 29739900 10.1128/mBio.00625-18 PMC5941073

[44] Malleret B , Li A , Zhang R , et al. Plasmodium vivax: restricted tropism and rapid remodeling of CD71‐positive reticulocytes. Blood. 2015;125(8):1314‐1324. 25414440 10.1182/blood-2014-08-596015 PMC4401350

[45] Baro B , Deroost K , Raiol T , et al. *Plasmodium vivax* gametocytes in the bone marrow of an acute malaria patient and changes in the erythroid miRNA profile. PLoS Negl Trop Dis. 2017;11(4):e0005365. 28384192 10.1371/journal.pntd.0005365 PMC5383020

[46] Silva‐Filho JL , Dos‐Santos JCK , Judice C , et al. Total parasite biomass but not peripheral parasitaemia is associated with endothelial and haematological perturbations in *Plasmodium vivax* patients. Elife. 2021;10:e71351. 34585667 10.7554/eLife.71351 PMC8536259

[47] Joice R , Nilsson SK , Montgomery J , et al. *Plasmodium falciparum* transmission stages accumulate in the human bone marrow. Sci Transl Med. 2014;6(244):244re5. 10.1126/scitranslmed.3008882 PMC4175394 25009232

[48] Belyaev NN , Brown DE , Diaz AIG , et al. Induction of an IL7‐R+c‐Kithi myelolymphoid progenitor critically dependent on IFN‐γ signaling during acute malaria. Nat Immunol. 2010;11(6):477‐485. 20431620 10.1038/ni.1869

[49] Furusawa JI , Mizoguchi I , Chiba Y , et al. Promotion of expansion and differentiation of hematopoietic stem cells by Interleukin‐27 into myeloid progenitors to control infection in emergency myelopoiesis. PLOS Pathog. 2016;12(3):e1005507. doi:10.1371/journal.ppat.1005507 26991425 PMC4798290

[50] Ghosh D , Brown SL , Stumhofer JS . IL‐17 promotes differentiation of splenic LSK—lymphoid progenitors into B cells following *Plasmodium yoelii* infection. J Immunol. 2017;199(5):1783‐1795. 28733485 10.4049/jimmunol.1601972 PMC5585076

[51] Ghosh D , Wikenheiser DJ , Kennedy B , et al. An atypical splenic B cell progenitor population supports antibody production during plasmodium infection in mice. J Immunol. 2016;197(5):1788‐1800. 27448588 10.4049/jimmunol.1502199 PMC4992648

[52] Belyaev NN , Biró J , Langhorne J , Potocnik AJ . Extramedullary myelopoiesis in malaria depends on mobilization of myeloid‐restricted progenitors by IFN‐γ induced chemokines. PLOS Pathog. 2013;9(6):e1003406. Available from:. doi:10.1371/journal.ppat.1003406 23762028 PMC3675198

[53] Gazzinelli RT , Hieny S , Wynn TA , Wolf S , Sher A . Interleukin 12 is required for the T‐lymphocyte‐independent induction of interferon gamma by an intracellular parasite and induces resistance in T‐cell‐deficient hosts. Proc Natl Acad Sci U S A. 2022;90(13):6115‐6119. 10.1073/pnas.90.13.6115 PMC46878 8100999

[54] Ehmen HG , Lüder CGK . Long‐term impact of *Toxoplasma gondii* infection on human monocytes. Front Cell Infect Microbiol. 2019;9:235. 31316920 10.3389/fcimb.2019.00235 PMC6611340

[55] Amikura T , Kikuchi T , Kato J , et al. Toxoplasmosis after allogeneic hematopoietic stem cell transplantation: impact of serostatus‐based management. Transpl Infect Dis. 2021;23(3):e13506. 33174304 10.1111/tid.13506

[56] Schwenk HT , Khan A , Kohlman K , et al. Toxoplasmosis in pediatric hematopoietic stem cell transplantation patients. Transplant Cell Ther. 2021;27(4):292‐300. 33840441 10.1016/j.jtct.2020.11.003

[57] Štajner T , Vujić D , Srbljanović J , et al. Risk of reactivated toxoplasmosis in haematopoietic stem cell transplant recipients: a prospective cohort study in a setting withholding prophylaxis. Clin Microbiol Infect. 2022;28(5):733.e1‐733.e5. 10.1016/j.cmi.2021.09.012 34555535

[58] Prestes DP , Mendes C , Batista MV , et al. A case‐series of toxoplasmosis in hematopoietic stem cell transplantation: still a concern for endemic countries. Bone Marrow Transplant. 2018;53(10):1336‐1339. 29703971 10.1038/s41409-018-0179-4

[59] Rauwolf KK , Floeth M , Kerl K , Schaumburg F , Groll AH . Toxoplasmosis after allogeneic haematopoietic cell transplantation—disease burden and approaches to diagnosis, prevention and management in adults and children. Clin Microbiol Infect. 2021;27(3):378‐388. 33065238 10.1016/j.cmi.2020.10.009

[60] Lopes CS , Silva TL , de Almeida JCN , Costa LVS , Mineo TWP , Mineo JR . Transmission of *Toxoplasma gondii* infection due to bone marrow transplantation: validation by an experimental model. Front Med. 2019;6:227. 10.3389/fmed.2019.00227 PMC6804406 31681783

[61] Chou DB , Sworder B , Bouladoux N , et al. Stromal‐derived IL‐6 alters the balance of myeloerythroid progenitors during *Toxoplasma gondii* infection. J Leukoc Biol. 2012;92(1):123‐131. doi:10.1189/jlb.1011527 22493080 PMC3382309

[62] Marins‐Dos‐Santos A , Ayres‐Silva J d P , Antunes D , et al. Oral *Trypanosoma cruzi* acute infection in mice targets primary lymphoid organs and triggers extramedullary hematopoiesis. Front Cell Infect Microbiol. 2022;24(12):301. 10.3389/fcimb.2022.800395 PMC8990980 35402296

[63] Cnops J , Bockstal V , De Trez C , Miquel MC , Radwanska M , Magez S . Curative drug treatment of trypanosomosis leads to the restoration of B‐cell lymphopoiesis and splenic B‐cell compartments. Parasite Immunol. 2015;37(9):485‐491. doi:10.1111/pim.12209 26072963

[64] Cnops J , De Trez C , Bulte D , Radwanska M , Ryffel B , Magez S . IFN‐γ mediates early B‐cell loss in experimental African trypanosomosis. Parasite Immunol. 2015;37(9):479‐484. 26079128 10.1111/pim.12208

[65] Obishakin E , de Trez C , Magez S . Chronic *Trypanosoma congolense* infections in mice cause a sustained disruption of the B‐cell homeostasis in the bone marrow and spleen. Parasite Immunol. 2014;36(5):187‐198. 24451010 10.1111/pim.12099

[66] Müller U , Schaub GA , Mossmann H , Köhler G , Carsetti R , Hölscher C . Immunosuppression in experimental chagas disease is mediated by an alteration of bone marrow stromal cell function during the acute phase of infection. Front Immunol. 2018;9:2794. 30619242 10.3389/fimmu.2018.02794 PMC6295583

[67] Marcondes MCG , Borelli P , Yoshida N , Russo M . Acute Trypanosoma cruzi infection is associated with anemia, thrombocytopenia, leukopenia, and bone marrow hypoplasia: reversal by nifurtimox treatment. Microbes Infect. 2000;2(4):347‐352. 10817635 10.1016/s1286-4579(00)00333-6

[68] Radwanska M , HTT N , Magez S . African trypanosomosis obliterates DTPa vaccine‐induced functional memory so that post‐treatment *Bordetella pertussis* challenge fails to trigger a protective recall response. Vaccines. 2021;9(6):603. 34200074 10.3390/vaccines9060603 PMC8230080

[69] De Beule N , Menu E , Bertrand MJM , et al. Experimental African trypanosome infection suppresses the development of multiple myeloma in mice by inducing intrinsic apoptosis of malignant plasma cells. Oncotarget. 2017;8(32):52016‐52025. 28881710 10.18632/oncotarget.18152 PMC5581009

[70] McSorley HJ , Hewitson JP , Maizels RM . Immunomodulation by helminth parasites: defining mechanisms and mediators. Int J Parasitol Pergamon. 2013;43:301‐310. 10.1016/j.ijpara.2012.11.011 23291463

[71] McSorley HJ , Maizels RM . Helminth infections and host immune regulation. Clin Microbiol Rev. 2012;25(4):585‐608. 23034321 10.1128/CMR.05040-11 PMC3485755

[72] Lenzi HL , Lenzi JA , Rosman FC , et al. Extramedullary hematopoiesis in murine schistosomiasis mansoni. Mem Inst Oswaldo Cruz. 1995;90(2):169‐177. 8531653 10.1590/s0074-02761995000200008

[73] Francisco JS , Terra MABL , Klein GCT , Dias de Oliveira BCEP , Pelajo‐Machado M . The hepatic extramedullary hematopoiesis during experimental murine *Schistosomiasis mansoni* . Front Immunol. 2022;13:955034. 36091027 10.3389/fimmu.2022.955034 PMC9453041

[74] Ishikawa N , Horii Y , Nawa Y . Reconstitution by bone marrow grafting of the defective protective capacity at the migratory phase but not at the intestinal phase of *Nippostrongylus brasiliensis* infection in W/Wv mice. Parasite Immunol. 1994;16(4):181‐186. doi:10.1111/j.1365-3024.1994.tb00338.x 8058356

[75] Babayan SA , Sinclair A , Duprez JS , Selman C . Chronic helminth infection burden differentially affects haematopoietic cell development while ageing selectively impairs adaptive responses to infection. Sci Rep. 2018;8(1):1‐12. 29491449 10.1038/s41598-018-22083-5 PMC5830876

[76] Goodridge HS , Marshall FA , Wilson EH , et al. In vivo exposure of murine dendritic cell and macrophage bone marrow progenitors to the phosphorylcholine‐containing filarial nematode glycoprotein ES‐62 polarizes their differentiation to an anti‐inflammatory phenotype. Immunology. 2004;113(4):491‐498. 15554927 10.1111/j.1365-2567.2004.01993.x PMC1782600

[77] Herbst T , Esser J , Prati M , et al. Antibodies and IL‐3 support helminth‐induced basophil expansion. Proc Natl Acad Sci U S A. 2012;109(37):14954‐14959. doi:10.1073/pnas.1117584109 22930820 PMC3443190

[78] Schneider E , Petit‐Bertron A‐F , Bricard R , et al. IL‐33 activates unprimed murine basophils directly in vitro and induces their In vivo expansion indirectly by promoting hematopoietic growth factor production. J Immunol. 2009;183(6):3591‐3597. 19684081 10.4049/jimmunol.0900328

[79] Siracusa MC , Saenz SA , Hill DA , et al. TSLP promotes interleukin‐3‐independent basophil haematopoiesis and type 2 inflammation. Nature. 2011;477(7363):229‐233. 21841801 10.1038/nature10329 PMC3263308

[80] Siracusa MC , Saenz SA , Tait Wojno ED , et al. Thymic stromal lymphopoietin‐mediated extramedullary hematopoiesis promotes allergic inflammation. Immunity. 2013;39(6):1158‐1170. 24332033 10.1016/j.immuni.2013.09.016 PMC3959827

[81] Saenz SA , Siracusa MC , Perrigoue JG , et al. IL25 elicits a multipotent progenitor cell population that promotes T H 2 cytokine responses. Nature. 2010;464(7293):1362‐1366. 20200520 10.1038/nature08901 PMC2861732

[82] Saenz SA , Siracusa MC , Monticelli LA , et al. IL‐25 simultaneously elicits distinct populations of innate lymphoid cells and multipotent progenitor type 2 (MPPtype2) cells. J Exp Med. 2013;210(9):1823‐1837. 23960191 10.1084/jem.20122332 PMC3754870

[83] Chenery AL , Antignano F , Hughes MR , Burrows K , McNagny KM , Zaph C . Chronic Trichuris muris infection alters hematopoiesis and causes IFN‐γ‐expressing T‐cell accumulation in the mouse bone marrow. Eur J Immunol. 2016;46(11):2587‐2596. doi:10.1002/eji.201646326 27594558

[84] Liu AY , Dwyer DF , Jones TG , et al. Mast cells recruited to mesenteric lymph nodes during helminth infection remain Hypogranular and produce IL‐4 and IL‐6. J Immunol. 2013;190(4):1758‐1766. 23319739 10.4049/jimmunol.1202567 PMC3563837

[85] Grencis RK , Else KJ , Huntley JF , Nishikawa SI . The in vivo role of stem cell factor (c‐kit ligand) on mastocytosis and host protective immunity to the intestinal nematode *Trichinella spiralis* in mice. Parasite Immunol. 1993;15(1):55‐59. 7679484 10.1111/j.1365-3024.1993.tb00572.x

[86] Rashidi NM , Scott MK , Scherf N , et al. In vivo time‐lapse imaging shows diverse niche engagement by quiescent and naturally activated hematopoietic stem cells. Blood. 2014;124(1):79‐83. 24850759 10.1182/blood-2013-10-534859 PMC4125355

[87] Inclan‐Rico JM , Hernandez CM , Henry EK , et al. *Trichinella spiralis*‐induced mastocytosis and erythropoiesis are simultaneously supported by a bipotent mast cell/erythrocyte precursor cell. PLoS Pathog. 2020;16(5):e1008579. 32421753 10.1371/journal.ppat.1008579 PMC7259795

[88] Henry EK , Sy CB , Inclan‐Rico JM , et al. Carbonic anhydrase enzymes regulate mast cell–mediated inflammation. J Exp Med. 2016;213(9):1663‐1673. doi:10.1084/jem.20151739 27526715 PMC4995079

[89] Gerbe F , Sidot E , Smyth DJ , et al. Intestinal epithelial tuft cells initiate type 2 mucosal immunity to helminth parasites. Nature. 2016;529(7585):226‐230. 26762460 10.1038/nature16527 PMC7614903

[90] Gracz AD , Samsa LA , Fordham MJ , et al. SOX4 promotes ATOH1‐independent intestinal secretory differentiation toward tuft and enteroendocrine fates. Gastroenterology. 2018;155(5):1508‐1523.e10. 30055169 10.1053/j.gastro.2018.07.023 PMC6232678

[91] Duckworth CA . Identifying key regulators of the intestinal stem cell niche. Biochem Soc Trans. 2021;49(5):2163‐2176. 34665221 10.1042/BST20210223 PMC8589435

[92] Lindholm HT , Parmar N , Drurey C , et al. BMP signaling in the intestinal epithelium drives a critical feedback loop to restrain IL‐13‐driven tuft cell hyperplasia. Sci Immunol. 2022;7(71):eabl6543. 35559665 10.1126/sciimmunol.abl6543 PMC7614132

[93] Drurey C , Lindholm HT , Coakley G , et al. Intestinal epithelial tuft cell induction is negated by a murine helminth and its secreted products. J Exp Med. 2022;219(1):e2021114. 10.1084/jem.20211140 PMC8597987 34779829

[94] Karo‐Atar D , Ouladan S , Javkar T , et al. Helminth‐induced reprogramming of the stem cell compartment inhibits type 2 immunity. J Exp Med. 2022;219(9):e20212311. doi:10.1084/jem.20212311 35938990 PMC9365672

[95] Divangahi M , Aaby P , Khader SA , et al. Trained immunity, tolerance, priming and differentiation: distinct immunological processes. Nat Immunol. 2020;22(1):2‐6. 10.1038/s41590-020-00845-6 PMC8020292 33293712

[96] Netea MG , Domínguez‐Andrés J , Barreiro LB , et al. Defining trained immunity and its role in health and disease. Nat Rev Immunology Nature Research. 2020;20:375‐388. 10.1038/s41577-020-0285-6 PMC7186935 32132681

[97] Netea MG , Joosten LAB , Latz E , et al. Trained immunity: a program of innate immune memory in health and disease. Science. 2016;352:427. 10.1126/science.aaf1098 PMC5087274 27102489

[98] Mitroulis I , Ruppova K , Wang B , et al. Modulation of myelopoiesis progenitors is an integral component of trained immunity. Cell. 2018;172(1–2):147‐161.e12. 29328910 10.1016/j.cell.2017.11.034 PMC5766828

[99] Kaufmann E , Sanz J , Dunn JL , et al. BCG educates hematopoietic stem cells to generate protective innate immunity against tuberculosis. Cell. 2018;172(1–2):176‐190.e19. 29328912 10.1016/j.cell.2017.12.031

[100] Chavakis T , Mitroulis I , Hajishengallis G . Hematopoietic progenitor cells as integrative hubs for adaptation to and fine‐tuning of inflammation. Nat Immunol. 2019;20:802‐811. 31213716 10.1038/s41590-019-0402-5 PMC6709414

[101] de Laval B , Maurizio J , Kandalla PK , et al. C/EBPβ‐dependent epigenetic memory induces trained immunity in hematopoietic stem cells. Cell Stem Cell. 2020;26(5):793. 32386557 10.1016/j.stem.2020.03.014

[102] Bomans K , Schenz J , Sztwiertnia I , Schaack D , Weigand MA , Uhle F . Sepsis induces a long‐lasting state of trained immunity in bone marrow monocytes. Front Immunol. 2018;9:2685. 30510555 10.3389/fimmu.2018.02685 PMC6254543

[103] Moorlag SJCFM , Rodriguez‐Rosales YA , Gillard J , et al. BCG vaccination induces long‐term functional reprogramming of human neutrophils. Cell Rep. 2020;33(7):108387. 33207187 10.1016/j.celrep.2020.108387 PMC7672522

[104] Van Der Meer JWM , Barza M , Wolff SM , Dinarello CA . A low dose of recombinant interleukin 1 protects granulocytopenic mice from lethal Gram‐negative infection. Proc Natl Acad Sci U S A. 1988;85:1620‐1623. 3125553 10.1073/pnas.85.5.1620 PMC279825

[105] Askenase MH , Han SJ , Byrd AL , et al. Bone‐marrow‐resident NK cells prime monocytes for regulatory function during infection. Immunity. 2015;42(6):1130‐1142. 26070484 10.1016/j.immuni.2015.05.011 PMC4472558

[106] Cunningham KT , Finlay CM , Mills KHG . Helminth imprinting of hematopoietic stem cells sustains anti‐inflammatory trained innate immunity that attenuates autoimmune disease. J Immunol. 2021;206(7):1618‐1630. 33579723 10.4049/jimmunol.2001225

